# Breast cancer-related occupational exposures facing immigrant women

**DOI:** 10.1038/s41370-025-00808-9

**Published:** 2025-10-20

**Authors:** K. E. Knox, J. L. Ohayon, Erin Carrera, R. A. Rudel, R. Morello-Frosch

**Affiliations:** 1 Silent Spring Institute, Newton, MA USA; 2 Department of Nursing, University of San Francisco, San Francisco, CA USA; 3 California Nurses for Environmental Health & Justice, Bolinas, CA USA; 4 School of Public Health, University of California, Berkeley, CA USA

## Abstract

**Background:**

Immigrants comprise roughly 14% of the U.S. population, and studies indicate that breast cancer increases among some immigrant groups after relocating to the U.S.

**Objective:**

We characterized exposures to breast cancer-relevant chemicals in jobs commonly occupied by U.S. immigrant women, aged 18–65.

**Methods:**

We analyzed data from the American Community Survey Public Use Microdata Sample to profile which occupations are most prevalent for immigrant women and integrated these results with data on occupational chemical exposures from the Women’s Occupations and Risk from Chemicals tool, which identifies occupations with probable and possible chemical exposures of relevance for breast cancer.

**Results:**

Immigrant women most commonly work as house cleaners, nurses, cashiers, janitors, and care aides, and comprise 71% of manicurists. We prioritize the occupations house cleaners and nurses for their combination of high potential exposures and the large number of immigrant women employed in these occupations. Chemicals of interest are those found in fragrances, and cleaning and maintenance products, including phthalates, antimicrobials, and alkylphenols. Many of these compounds are mammary gland carcinogens and developmental toxicants, and/or endocrine disruptors.

**Impact:**

- There are few studies of breast cancer-relevant chemical exposures for most occupations, including those heavily represented by immigrant women. By identifying jobs that employ large numbers of immigrant women and are associated with a high likelihood of exposure to potential breast carcinogens, we inform future research on breast cancer-relevant exposures and opportunities for preventative exposure reduction. We also show that immigrant women with lower levels of education and English fluency work in occupations with more potential for harmful chemical exposures.

## Introduction

In the United States, breast cancer is the most common cancer diagnosis and the second leading cause of cancer death among women [1]. Comparisons of breast cancer rates between first- or second-generation immigrants in the U.S. versus those who have been in the U.S. longer have identified trends that indicate an important role of environment and behavioral changes on breast cancer risk. First-generation immigrants comprise just under 14% of the population in the United States [2]. Several studies found that U.S.-born women had higher rates of breast cancer than their immigrant counterparts [3–6], and that disease rates rise for some immigrant groups after relocating to the U.S., specifically in terms of longer time spent in the U.S. and across successive generations [3, 4, 6]. However, more recent studies of Asian American women in California have found higher breast cancer rates among foreign-born women than their U.S.-born counterparts [7, 8], and that the positive association between breast cancer rates and time spent in the U.S. only holds for younger women [8].

These shifting patterns, with higher risk in immigrant women in more recent years compared to their U.S.-born counterparts, suggests that the previously hypothesized lower-risk profile of recent immigrants (for example, due to living in enclaves with access to culturally and linguistically appropriate resources, services, and community support) [5] may be outweighed by environmental and occupational risk factors encountered in the United States and that may be higher for immigrant women compared to their U.S.-born counterparts. Such environmental factors may include changes in diet, access to health care, as well as housing and working conditions [9]. In addition, chemical exposures can raise breast cancer risk [10, 11]. Air pollution, for example, has been associated with increased breast cancer [10]. Studies indicate that workplace exposures influence breast cancer risk. This includes studies showing higher breast cancer risk associated with working in nursing and other health-related occupations, cleaning, agriculture, metalworking, and manufacturing of plastics, rubber, automobile parts, and textiles [10, 12–14]. In addition, many chemicals that have not been studied in humans have been shown in animal studies to increase the risk of mammary tumors [11, 15]. Thus, women exposed to these chemicals in the workplace may also experience elevated breast cancer risk.

We investigated workplace exposures of immigrant women in the U.S. to characterize where they may experience exposures to breast cancer-relevant chemicals (BCRCs). We hypothesize that occupations with high proportions of immigrant women often entail exposures to BCRCs. Specifically, we used publicly available data to answer two key questions: (1) In which U.S. occupations do immigrant women work? (2) What BCRC exposures do workers in these occupations likely face? Identifying settings that may increase breast cancer risk will shape further studies and actions to reduce harmful exposures.

## Methods

### Data

We used the 2019 American Community Survey (ACS), 1-year Public Use Microdata Sample (PUMS), to identify where immigrant workers were employed [16]. The original data file contained 3,239,553 observations. We subset this file to females ages 18–65 who were employed and had non-missing occupational data (n = 820,540). Of these individuals, 116,013 were born outside the U.S.

We then linked occupations that employed the most immigrant women with exposure data collected by the California Breast Cancer Research Program-funded Women’s Occupations and Risk from Chemicals (WORC) Project [17], which identifies occupations with chemical exposures relevant to breast cancer. The set of 1082 chemicals that may increase breast cancer risk was compiled by WORC from two published articles identifying chemicals that increase mammary tumors or alter mammary gland development, and the Endocrine Disruption Exchange (TEDX) database of endocrine-disrupting chemicals [18]. WORC combined chemicals into groups (e.g., antimicrobials, phthalates, cleaning and maintenance products). To create a job exposure matrix for these chemicals, industrial hygienists classified occupational groups as having probable, possible, or unlikely exposures to these groups of chemicals. A probable exposure was defined as exposure due to direct use or working in the presence of a chemical. A possible exposure reflected uncertainty either due to variability in chemical composition of common materials used in a job or variability of activities and associated exposures that may be part of that job category [18]. The WORC project classified 145 occupational categories representing 84% of the California female workforce [18]. Some occupations where immigrant women make up a large percentage of the workforce were not classified in the WORC tool, including interpreters, tailors, and gambling services workers.

To quantify the WORC data, we assigned a score of “1” to each unlikely exposure, “3” to each possible exposure, and “5” to each likely exposure. We then computed a raw exposure score for each occupation by summing the exposure scores across all chemical groups and then dividing the sum by 24 (the total number of chemical groups). So, for example, the occupation “management analysts”, which has unlikely exposures to all 24 chemical groups, received a score of 1, whereas the occupation “manicurists”, which had probable exposures to 4 chemical groups and possible exposures to 6 chemical groups, received a score of 2.17 (calculated as (4*5 + 6*3 + 14*1)/24).

We primarily considered the most prevalent occupations for immigrant women in the U.S. to be those 20 occupations that employ the largest absolute number of immigrant women. As a secondary analysis, we also subset to only those occupations that are in the upper half of the distribution in terms of total number of women employed, and then computed the percentage of the female workforce that was comprised of immigrants. We again selected the top 20 occupations, this time in terms of those that employed the most immigrant women in terms of percent of the total female workforce. We linked the identified occupations to the WORC data to identify probable and possible BCRC exposures associated with these occupations.

We also stratified the occupational data by demographics, including global region of birth, English-speaking ability, educational attainment (completed high school or not), and percent of life spent in the U.S. For each subgroup, we selected occupations that employed the most immigrant women workers in terms of number of workers and then linked the identified occupations to the WORC data.

To truly understand those occupations that are the highest priority in terms of the occupational exposures faced by immigrant women, we computed a weighted exposure score for each occupation, in which we multiplied the raw exposure score for each occupation by a weight, calculated as the number of immigrant women employed in that occupation divided by the total number of immigrant women across all occupations. We listed the 20 occupations that have the highest weighted exposure scores.

## Results

**Fig. 1:**
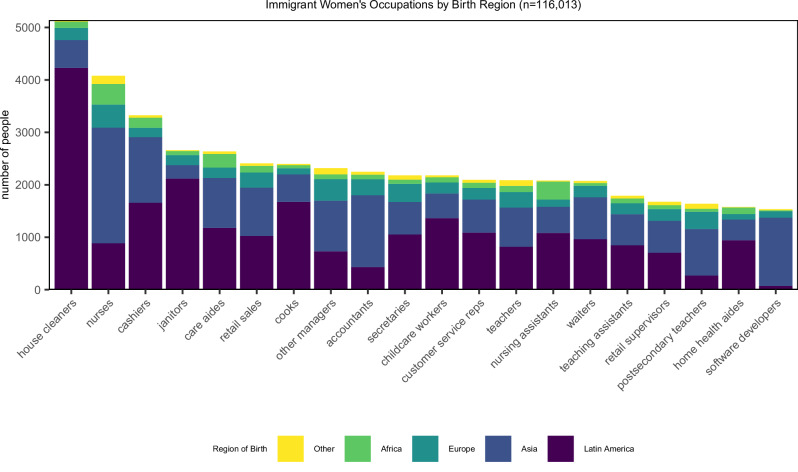
Most prevalent occupations of U.S. Immigrant Women Workers by Global Region of Birth. Sample includes 116,013 women ages 18–65 born outside the U.S. from the 2019 PUMS file. “Other” includes Canada, Oceania, and at sea.

The most prevalent U.S. occupations for immigrant women (in terms of number of people in our sample working in the occupation) were house cleaners, nurses, cashiers, janitors, and care aides (Fig. 1). Forty-four percent of the immigrant women in our sample were born in Latin America, 37% were born in Asia, 11% were born in Europe, 5% were born in Africa, and the remaining 3% were born elsewhere (Canada, Oceania or at sea). Common occupations varied by region of birth – for women born in Latin America, house cleaning was the most prevalent occupation, while nursing was the most prevalent for women born in Africa, Asia, and Europe. When we looked at occupations with the highest percentage of female immigrant workers, we found that 71% of female manicurists are immigrants, followed by agricultural graders/sorters (58%), software developers (45%), interpreters (41%), house cleaners (41%), and sewing machine operators (41%) (Table S1).

**Fig. 2:**
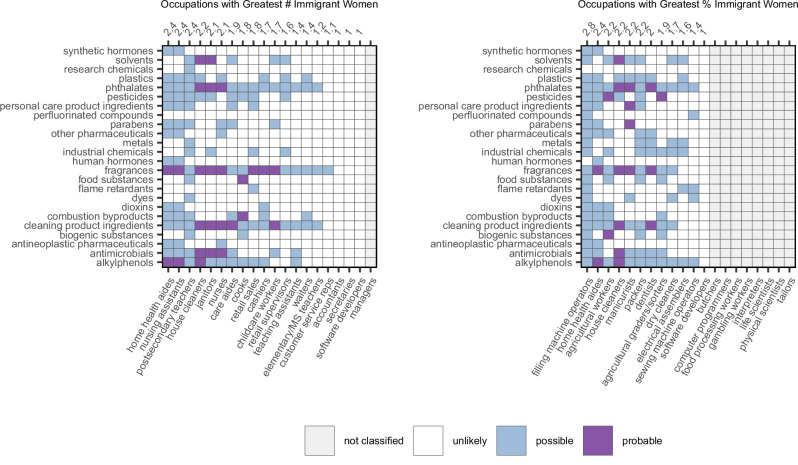
Left panel shows probable and possible chemical exposures posed by the occupations that employ the greatest number of immigrant women in the U.S., while right panel shows the probable and possible exposures posed by the U.S. occupations that employ the greatest percentage of immigrants (as a percentage of the total female workers). Purple boxes indicate probable chemical exposures, and blue boxes indicate possible chemical exposures. Numbers across the top axes indicate raw exposure scores for each occupation, rounded to the nearest tenth. Managerial occupations, interpreters, gambling workers, tailors, life scientists, physical scientists, butchers, computer programmers, and food processing workers were not classified in the WORC data.

Combining these highlighted occupations with the WORC data on probable and possible chemical exposures, we found that amongst those occupations that employed the largest number of immigrant women, home health aides, nursing assistants, and postsecondary teachers tied for the highest raw exposure score (2.4), followed by house cleaners (2.2), janitors (2.1), and nurses (2.1) (Fig. 2). When we weighted the raw exposure scores to account for the number of immigrant women working in each occupation, house cleaners had the highest score, followed by nurses (Table S2). House cleaners (the most prevalent occupation for immigrant women) were associated with the highest number of probable chemical exposures that include six groups of breast cancer-relevant chemicals: alkylphenols, antimicrobials, cleaning and maintenance product ingredients, fragrances, phthalates, and solvents (Fig. 2). House cleaners also had possible exposures to plastics and pesticides. Nurses (the second most prevalent occupation for immigrant women) had probable exposures to four groups of breast-cancer relevant compounds: antimicrobials, cleaning and maintenance product ingredients, fragrances, and phthalates. Nurses also had possible exposures to five additional chemical groups: plastics, parabens, other pharmaceuticals, antineoplastic pharmaceuticals, and alkylphenols.

Fragrance chemicals were a source of probable exposures for many of the occupations with high prevalence of immigrant women (8 probable exposures), followed by chemicals found in cleaning products (5 probable exposures; Fig. 2).

Looking at potential chemical exposures for women who work in occupations that employ a high percentage of immigrant women (Fig. 2), filling machine operators had the highest raw exposure score (2.8). Filling machine operators run machines to prepare industrial or consumer products for storage or shipment (including cannery workers who pack food products); for this occupation, more than a third of female workers are immigrants, and our analysis showed possible exposures to 22 different chemical groups relevant to breast cancer. Following filling machine operators, home health aides had a raw exposure score of 2.4. Agricultural workers, house cleaners, manicurists, and packers tied for third place with a raw exposure score of 2.2

**Fig. 3:**
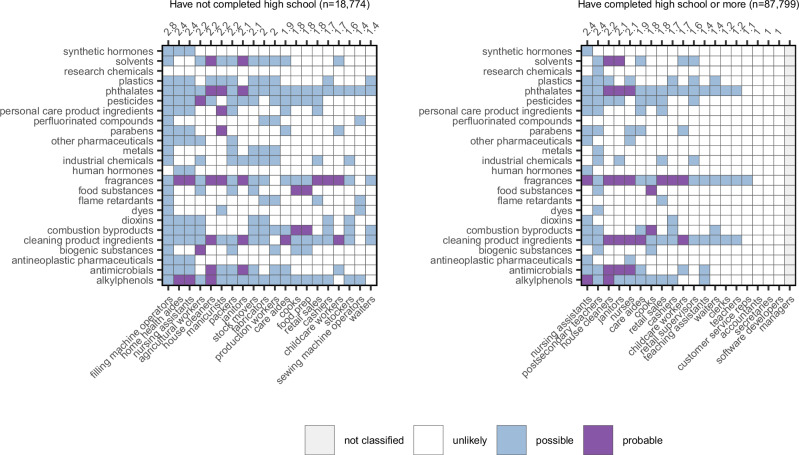
Probable and possible chemical exposures posed by U.S. occupations that employ the greatest number of immigrant women, broken out by educational attainment. Purple boxes indicate probable chemical exposures, and blue boxes indicate possible chemical exposures. Numbers across the top axes indicate raw exposure scores for each occupation, rounded to the nearest tenth. Managerial occupations were not classified in the WORC data.

Analyzing the data by other demographic characteristics (Figs. 3 and S1–S3), we found that higher educational attainment and better English-speaking ability were associated with access to occupations with minimal chemical exposures (such as accountants, customer service representatives, software developers) (Figs. 3 and S2). Immigrant women who have spent less than a quarter of their life in the U.S. face greater potential occupational chemical exposures than those who have spent more than three-quarters of their life in the U.S. (Fig. S3).

## Discussion

Immigrant women, especially those with lower levels of educational attainment and limited English-speaking ability, are likely exposed to numerous BCRCs at work. While there are limited studies on how environmental chemical exposures might increase breast cancer risk for immigrants, our results align with prior findings that lower educational attainment is associated with higher potential for occupational exposures to chemical hazards [19]. Studies have also shown that compared to their U.S.-born counterparts, immigrant women face higher environmental chemical exposures for different compound classes, including metals, phthalates, polybrominated diphenyl ethers (PDBEs), and pesticides [20]. While research gaps remain, immigrant workers can have more occupational exposures than U.S.-born workers [19]. In addition, immigrant women may face unique hurdles in avoiding occupational exposures, including an inability to challenge unsafe working conditions due to precarious employment, language barriers, undocumented status, and structural racism; immigrant women may also have less access to culturally and linguistically appropriate job-related health and safety information [21, 22].

In this paper, we highlight nurses (and the closely related occupational categories of nursing assistants and home health aides) as prevalent occupations for immigrant women that are associated with numerous BCRC exposures. Prior work has found that nurses have higher rates of breast cancer than the general population [23–25]. Additional work is suggestive that nurses face occupational exposures to breast cancer-relevant chemicals [26].

We also highlight the occupations of house cleaners, agricultural workers, and manicurists. Prior work has found women working in agriculture have elevated breast cancer risk [14, 27] and that women working in agriculture face potential reproductive hazards [28]. Other work has documented potential breast cancer-relevant exposures to nail salon workers, including to VOCs [29] and phthalates [30], as well as to house cleaners [31, 32].

An important limitation to this analysis is that being exposed to the greatest number of BCRCs does not necessarily translate into the highest exposures. For example, while agricultural workers might be exposed to fewer types of chemicals than nursing assistants, they may have higher levels and durations of exposure. A related limitation is that some chemicals may be more toxic than others with respect to breast cancer risk. For example, chemicals shown to cause mammary gland tumors in experimental animal studies—especially if they damage DNA—may be more likely to increase breast cancer risk compared with weaker endocrine-disrupting chemicals. In addition, for endocrine-disrupting chemicals in particular, exposure may be more problematic during critical windows of susceptibility, such as puberty and pregnancy. Furthermore, exposures vary depending on the job type within an occupational group (for example, crop harvesting versus pesticide spraying in agricultural work) [33]. Although we look at the probability of exposure to BCRCs for each occupation group, we do not have information on exposure levels or the intensity of these exposures between different occupations. Some occupations end up having a high raw exposure score due to a large number of possible chemical exposures (for example, postsecondary teachers and filling machine operators). However, workers in these occupations can work in many different types of settings; an English teacher likely has very different exposures than a chemistry teacher, and consequently, some workers in these occupations may have just a small subset of the possible exposures.

There are also important limitations to the datasets that we used in our analysis. The WORC database was built with a limited list of chemicals, and many other BCRCs were not included because they had not yet been identified. For example, our team recently published a novel list of 920 breast cancer-relevant chemicals [11], and only 331 of them were included in the WORC job exposure matrix; it is thus not known which occupations have exposures to these newly-identified breast-cancer relevant chemicals. Another limitation of the WORC data is that there are some occupations that are substantial employers of immigrant workers in the U.S. that are not classified in the WORC data. Our analysis illuminates that many occupations have poorly characterized chemical exposures, especially those most relevant for immigrants, which is a barrier to studying health outcomes or designing exposure reduction strategies.

Despite these limitations, our novel screening approach identified several occupations with a high prevalence of immigrant women who likely face BCRC exposures. Many of these chemical exposures have also been linked to other common chronic diseases, including other cancers. Results can inform future research on breast cancer risk factors among immigrant women by emphasizing an understudied and actionable area—occupational exposures.

## Supplementary information

Supplemental Material

## Data Availability

All data used in this analysis is publicly available online. The American Community Survey PUMS data is found here: https://www.census.gov/programs-surveys/acs/microdata.html. The WORC data is found here: https://cbcrp.org/worker-exposure/
