# Supplementary material for: Breast cancer-related occupational exposures facing immigrant women
Source: J Expo Sci Environ Epidemiol. 2025 Oct 20;36(2):355–60. doi: 10.1038/s41370-025-00808-9 (PMC12960209; doi:10.1038/s41370-025-00808-9)
Supplement: Supplementary file 1 — Supplemental Material [file 41370_2025_808_MOESM1_ESM.pdf]

Supplemental Material

Breast Cancer Related Occupational Exposures Facing Immigrant Women

K.E. Knox[1*], J.L. Ohayon[1], R.A. Rudel[1], R. Morello-Frosch[2]

[1] Silent Spring Institute, Newton, MA 02460 USA

[2] School of Public Health, University of California, Berkeley, CA 94720 USA

*Email: knox@silentspring.org

Contents

Table S1: Most Prevalent U.S. Occupations by % of female workers that are immigrants

Table S2: U.S. Occupations with Highest Weighted Exposure Score

Figure S1: Probable and possible chemical exposures posed by the occupations that employ the greatest number of immigrant women, broken out by world area of birth.

Figure S2: Probable and possible chemical exposures posed by the occupations that employ the greatest number of immigrant women, broken out by English-speaking ability.

Figure S3: Probable and possible chemical exposures posed by the occupations that employ the greatest number of immigrant women, broken out by portion of life spent in the U.S.

Table S1: Most Prevalent Occupations in the U.S. by % of Female Workers that are Immigrants

**Occupation**	**% Immigrant**	**Total # of Women Workers**
Manicurists	71%	2,031
Agricultural Graders/Sorters	58%	434
Software Developers	45%	3,407
Interpreters	41%	836
House cleaners	41%	12,568
Sewing machine operators	41%	1,278
Gambling workers	40%	512
Tailors	40%	570
Agricultural workers	40%	2,432
Life scientists	39%	900
Packers	38%	2,964
Physical scientists	37%	1,583
Electrical assemblers	37%	673
Filling machine operators	36%	1,474
Dentists	34%	626
home health aides	33%	4,713
Dry cleaners	31%	1,056
Butchers	31%	564
Computer programmers	30%	858
Food processing workers	30%	521

The table above shows the most prevalent occupations by the percentage of female workers that are immigrants. Sample includes 116,013 females ages 18-65 born outside the U.S. and 704,527 females ages 18-65 born in the U.S. from the 2019 PUMS file.

Table S2: U.S. Occupations with Highest Weighted Exposure Score

Occupation	Raw Exposure Score	Weighted Exposure Score	Total # of Immigrant Women Workers
House cleaners	2.2	0.098	5143
Nurses	2.1	0.074	4079
Cashiers	1.7	0.049	3327
Janitors	2.1	0.048	2661
Personal care aides	1.9	0.043	2637
Nursing assistants	2.4	0.043	2081
Retail salespersons	1.8	0.037	2409
Cooks	1.8	0.037	2398
Postsecondary teachers	2.4	0.034	1639
Home health aides	2.4	0.033	1577
Childcare workers	1.7	0.032	2180
Manicurists	2.2	0.027	1444
Waitstaff	1.4	0.025	2073
Retail sales supervisors	1.6	0.023	1678
Hair stylists	2.3	0.022	1112
Teaching assistants	1.4	0.022	1791
Elementary/MS teachers	1.2	0.022	2087
Hand packers	2.2	0.021	1119
Customer service reps	1.1	0.020	2095
Accountants	1.0	0.019	2249

The table above shows the 20 U.S. occupations with the highest weighted exposure score. To calculate the exposure score, we assigned a score of “1” to each unlikely exposure, “3” to each possible exposure, and “5” to each likely exposure. We computed the raw exposure score for each occupation as the sum of the exposure scores across all chemical groups, divided by 24 (the total number of chemical groups). Raw exposure scores shown in the table above are rounded to the nearest tenth for readability. The weighted exposure score is the raw exposure score for each occupation, multiplied by the number of immigrant women employed in that occupation, divided by the total number of immigrant women across all occupations. Sample includes 116,013 females ages 18-65 born outside the U.S. and 704,527 females ages 18-65 born in the U.S. from the 2019 PUMS file.

Figure S1

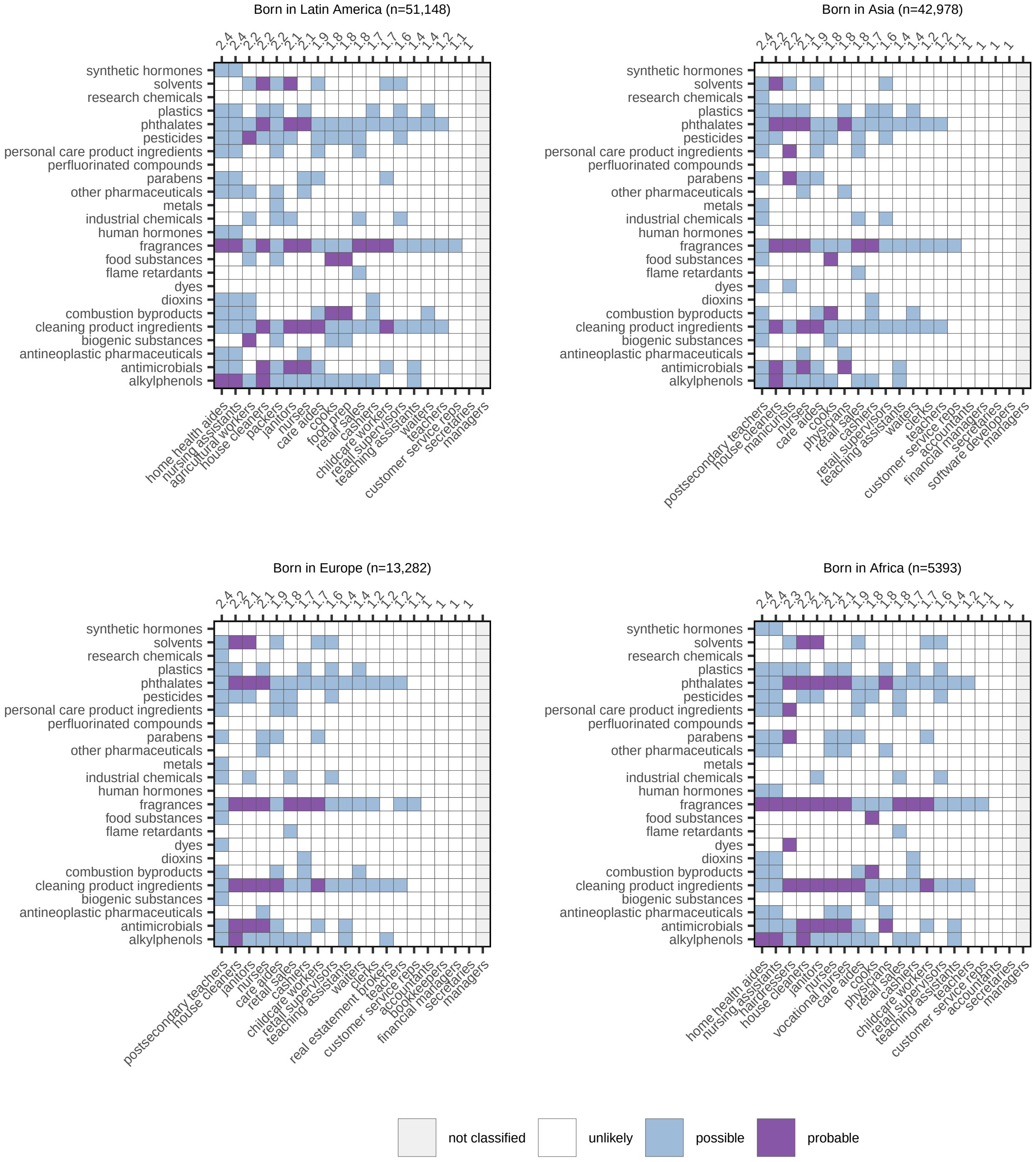

Probable and possible chemical exposures posed by the occupations that employ the greatest number of immigrant women, broken out by world area of birth. Managerial occupations were not classified in the WORC data. Numbers across the top axes indicate raw exposure scores for each occupation.

Figure S2

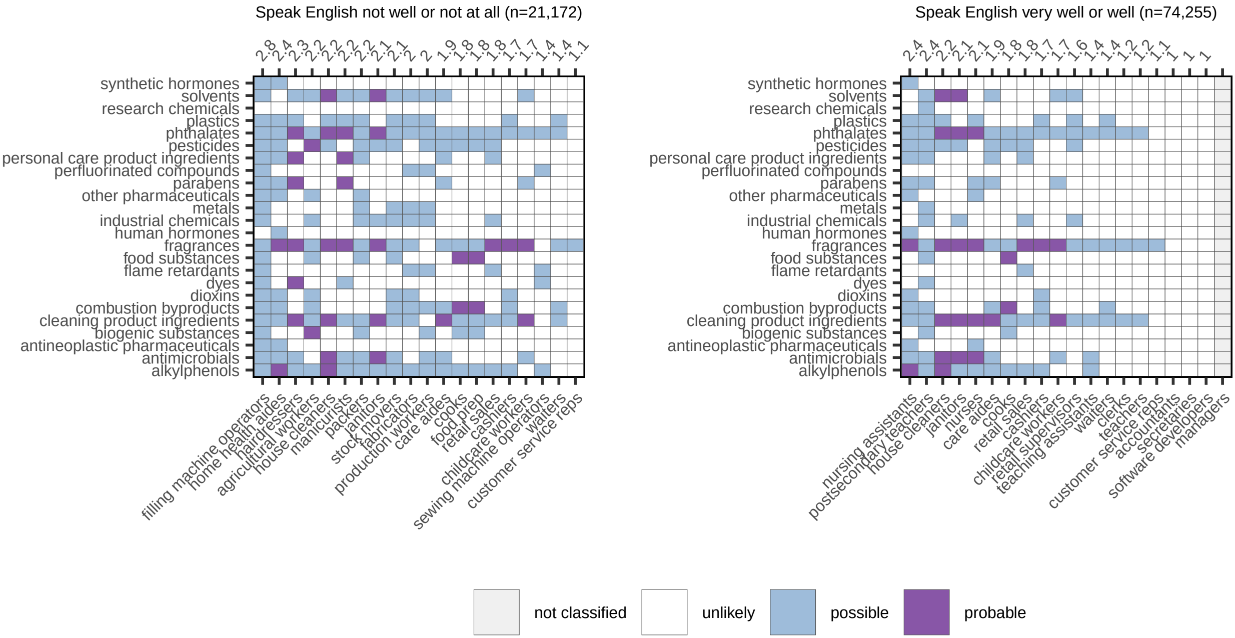

Probable and possible chemical exposures posed by the occupations that employ the greatest number of immigrant women, broken out by English−speaking ability. Managerial occupations were not classified in the WORC data. Numbers across the top axes indicate raw exposure scores for each occupation.

Figure S3

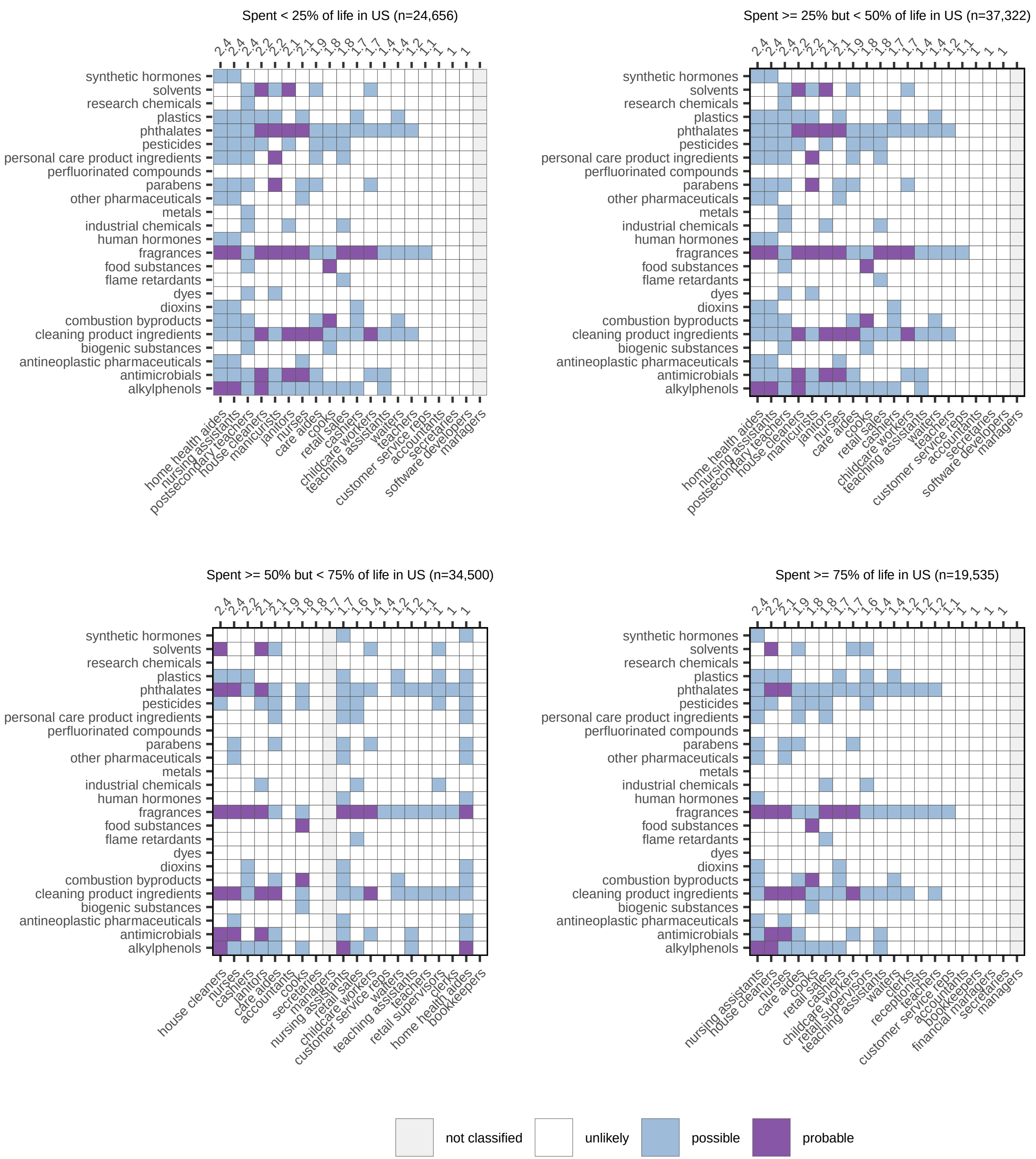

Probable and possible chemical exposures posed by the occupations that employ the greatest number of immigrant women, broken out by portion of life spent in the U.S. Managerial occupations were not classified in the WORC data. Numbers across the top axes indicate raw exposure scores for each occupation.
